# Endometriosis as a cause of reduced IVF success rates: guilty beyond reasonable doubt?

**DOI:** 10.5935/1518-0557.20260026

**Published:** 2026

**Authors:** Melissa Cavagnoli, Maite del Collado, Vitória G. Ambrósio, Gustavo Telles, Maria Augusta Tamm, Ana Micucci, Edson Borges

**Affiliations:** 1 Clínica Hope Reprodução Humana, São Paulo, São Paulo, Brazil; 2 Science For Everymind, São Paulo, São Paulo, Brazil; 3 Fertility - FertGroup Medicina Reprodutiva, São Paulo, São Paulo, Brazil

**Keywords:** endometriosis, in vitro fertilization, oocytes, ovarian reserve, endometrial receptivity, assisted reproductive technology

## Abstract

Endometriosis is a chronic inflammatory disease that affects around 10% of women of reproductive age and is frequently associated with infertility. Despite its high prevalence in assisted reproductive technology (ART) cycles, the exact mechanisms by which endometriosis may impair in vitro fertilization (IVF) outcomes remain under debate. This integrative narrative review critically examines the evidence on how endometriosis may affect oocyte quality and competence, ovarian reserve, and endometrial receptivity. While several studies have suggested molecular and cellular alterations in the follicular microenvironment and endometrial environment, population-based registry analyses have reported conflicting results regarding their clinical impact. Instead, the most reproducible negative impact appears to be a reduction in the number of oocytes retrieved, particularly in cases involving endometriomas or after ovarian surgery. Similarly, while endometrial alterations have been observed in women with endometriosis, large registry-based studies of donor oocyte cycles suggest only a marginal impairment of endometrial receptivity, if any. Taken together, reasonable doubt remains regarding the extent to which endometriosis influences fertility outcomes in IVF cycles.

## INTRODUCTION

Endometriosis is a chronic, benign, estrogen-dependent inflammatory disease characterized by the presence of endometrial-like tissue outside the uterine cavity. Its pathogenesis is most commonly attributed to the retrograde menstruation of viable endometrial cells into the pelvic cavity. Endometriosis affects 5 to 10% of reproductive-age women worldwide and, to date, there is no medical or surgical cure (Ghiasi *et al.*, 2020; As-Sanie *et al.*, 2025; La Marca *et al.*, 2025).

Endometriosis is classified into four subtypes, which may coexist and have implications for diagnosis and treatment: (1) superficial peritoneal endometriosis, involving lesions located on the peritoneal surface; (2) deep endometriosis, characterized by lesions infiltrating pelvic structures such as the uterosacral ligaments, bowel, or urinary tract; (3) endometriomas, cystic ovarian formations lined by endometrial tissue; and (4) extrapelvic endometriosis, involving sites beyond the pelvis, including the diaphragm, abdominal wall, and central nervous system (As-Sanie *et al.*, 2025). Moreover, the American Society for Reproductive Medicine (ASRM) classifies endometriosis into four stages (Stages I-IV) based on the extent, location, depth of the lesions, and the presence of adhesions (Canis *et al.*, 1997).

Dysmenorrhea, nonmenstrual pelvic pain, and dyspareunia are the most common symptoms of endometriosis and have frequently been associated with infertility (As-Sanie *et al.*, 2025). Approximately 30-50% of women with endometriosis are estimated to experience infertility (Meuleman *et al.*, 2009; Evans & Decherney, 2017). Moreover, according to a recent systematic review, the prevalence of endometriosis among women diagnosed with unexplained infertility is approximately 44% (Van Gestel *et al.*, 2024). As a result, many couples pursue assisted reproductive technologies (ART) for infertility treatment, with endometriosis representing approximately 10% of the indications for in vitro fertilization (IVF) (Somigliana *et al.*, 2017).

This review critically examines the evidence on the relationship between endometriosis and infertility, aiming to challenge prevailing assumptions and encourage a more nuanced understanding of its impact on oocyte quality and competence, ovarian reserve, and endometrial receptivity. This article was conceived as an interpretative narrative review aimed at conceptually integrating molecular, cellular, and clinical evidence related to endometriosis-associated infertility. The literature search was conducted in PubMed for studies published up to January 2025, using combinations of the following keywords: endometriosis, endometrioma, deep endometriosis, oocyte quality, oocyte competence, embryo quality, ovarian reserve, endometrial receptivity and IVF outcomes. References were selected based on their scientific relevance and contribution to conceptual and mechanistic discussion.

## FROM ASSUMPTION TO INTERROGATION: TRACING THE DEBATE ON ENDOMETRIOSIS AND INFERTILITY

Several factors have been proposed as contributors to endometriosis-related infertility, including: (i) dyspareunia, (ii) mechanical factors such as adhesions or anatomical distortion, (iii) diminished ovarian reserve due to endometrioma, (iv) reduced oocyte quality, (v) sperm dysfunction, (vi) impaired tubo-ovarian interaction or embryo transport difficulties, (vii) disrupted ovulation, and (viii) endometrial receptivity dysfunction, among others. Many of these potential causes of infertility in endometriosis are associated with the systemic inflammatory environment induced by the disease (Bonavina & Taylor, 2022).

Reactive oxygen species, immune system dysregulation, and disruption of cellular architecture due to impaired differentiation, proliferation, and apoptosis are critical mechanisms that could negatively impact the reproductive environment (Simopoulou *et al.*, 2021).

The direct causal relationship between tubal dysfunction secondary to adhesions in endometriosis and infertility has been extensively documented and considered irrefutable within the scientific community. Likewise, dyspareunia attributed to endometriosis is widely acknowledged as a critical factor compromising sexual function and quality of life. However, outside the context of tubal dysfunction and pain, the association between endometriosis and infertility has been called into question, as some authors have highlighted the lack of supporting evidence (Ata & Somigliana, 2024).

Generating high-quality scientific evidence on the true impact of endometriosis on fertility remains a major challenge. Several methodological limitations complicate the interpretation of existing studies. First, there is considerable heterogeneity in disease stage, lesion phenotype, and prior surgical history, which makes patient populations difficult to compare. Second, isolating the specific contribution of endometriosis itself is often problematic, as adenomyosis frequently coexists and may independently influence reproductive outcomes. Moreover, differences in the type, extent, and timing of surgical interventions can significantly affect ovarian reserve and endometrial function, further confounding results. Finally, when considering evidence derived from ART cycles, additional variability arises from the diversity of treatment strategies applied at different stage of the cycle (before ovarian stimulation or prior to embryo transfer). Together, these factors make it difficult to accurately determine the extent to which endometriosis itself contributes to infertility.

## OOCYTE QUALITY *VS*. COMPETENCE: WHAT DOES ENDOMETRIOSIS REALLY AFFECT?

**Figure 1:**
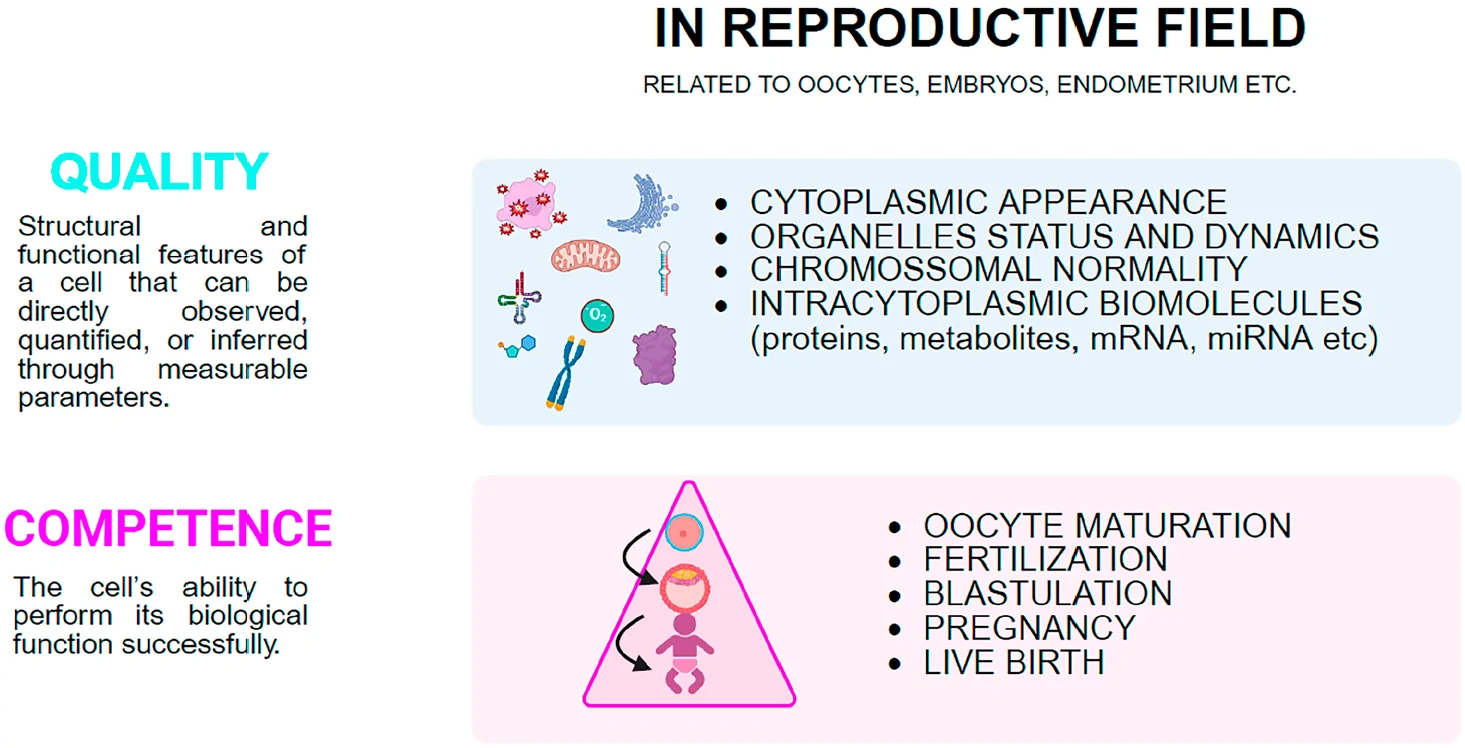
Conceptual distinction between cellular quality and competence in reproduction. Quality reflects intrinsic cellular features, including nuclear and cytoplasmic morphology, organelle integrity, chromosomal stability, and key molecular components. Competence denotes the functional capacity to achieve key developmental endpoints

In the scientific literature, the concepts of quality and competence are often confused or used interchangeably. However, although related, these terms have distinct definitions and encompass independent characteristics that are essential to consider (Figure 1). Oocyte competence refers to the oocyte’s functional ability to successfully undergo and complete critical reproductive processes, including maturation, fertilization, early embryonic development, and progression to a viable blastocyst (Rienzi *et al.*, 2012; Palomba *et al.*, 2017). Thus, a competent oocyte is defined by its ability to successfully complete these critical developmental milestones. In contrast, oocyte quality is generally associated with the morphological and cytoplasmic features of the oocyte, observable structural and functional characteristics of a cell, including structural integrity and organelle distribution (Rienzi *et al.*, 2012; Palomba *et al.*, 2017). Distinguishing between these concepts is crucial, as certain pathological conditions, including endometriosis, may impact oocyte quality (e.g., altering morphology or specific organelle patterns) without necessarily compromising oocyte competence, such as its ability to reach the blastocyst stage. This conceptual distinction is frequently neglected in the literature, where the terms “quality” and “competence” are often used interchangeably, leading to misinterpretation of findings and clinical implications.

Endometriosis is associated with alterations in the follicular microenvironment that may impair cumulus cell function and compromise oocyte quality at both the cellular and molecular levels. The importance of the follicular environment for ensuring oocyte quality and developmental competence is well established. Cumulus cells, which are directly exposed to this environment, support oocyte growth throughout folliculogenesis via transzonal projections, extracellular vesicles, and gap junctions. This intimate communication enables the regulation of oocyte meiotic reactivation, the transfer of key metabolic substrates such as pyruvate, and the delivery of molecules essential for maintaining intracellular homeostasis (Del Collado *et al.*, 2018). Consequently, it is plausible that disruptions to the follicular environment may adversely affect oocyte quality and competence.

Endometriosis has been associated with decreased levels of estrogen and testosterone and increased levels of progesterone in the follicular fluid. These alterations are thought to result from local inflammation and impaired steroidogenesis in theca and granulosa cells, leading to reduced estradiol and testosterone production. Conversely, the elevated progesterone levels are likely a consequence of premature luteinization and altered steroid metabolism within the inflammatory microenvironment (Fan *et al.*, 2023). Additionally, studies have demonstrated enhanced activation of apoptotic pathways and a higher number of apoptotic bodies in follicular cells of patients with endometriosis (Demirel *et al.*, 2001; Goud *et al.*, 2014; Sanchez *et al.*, 2016). A larger prospective study involving patients with stage III and IV endometriosis identified distinct profiles of pro-inflammatory and anti-inflammatory cytokine signaling within the follicular fluid (Singh *et al.*, 2016). Endometriosis has also been linked to increased endoplasmic reticulum stress and oxidative stress in granulosa and cumulus cells (Scutiero *et al.*, 2017; Kunitomi *et al.*, 2020; Lin *et al.*, 2020).

At the oocyte level, transcriptomic profiling of cumulus cells from nine patients with stage I-II endometriosis identified 26 differentially expressed genes implicated in the regulation of inflammatory and immune pathways when compared to controls (Da Luz *et al.*, 2022). Additionally, transcriptomic analysis of individual metaphase II (MII) oocytes from seven patients with endometriosis, contrasted with oocytes from healthy donors, revealed widespread gene expression alterations independent of ovarian laterality, affecting pathways involved in steroid metabolism, oxidative stress response, and cell cycle regulation (Ferrero *et al.*, 2019). Endometriosis has also been associated with mitochondrial dysfunction and morphological changes (Xu *et al.*, 2015), as well as disruptions in cortical granule dynamics and meiotic spindle integrity within oocytes during an in vitro maturation (IVM) experiment (Goud *et al.*, 2014).

Beyond cellular and molecular alterations, endometriosis has further been related to compromised oocyte quality, including an increased proportion of oocytes presenting morphological abnormalities (Shebl *et al.*, 2017; Kasapoglu *et al.*, 2018; Robin *et al.*, 2021). Notably, the influence on oocyte quality could be affected by endometriosis stage, as indicated by Shebl et al. (2017), who reported a much severe phenotype regarding oocyte morphology in stage IV. Moreover, a retrospective cohort study of more than 6,000 oocytes concluded that besides a decrease in the number of recovered oocytes and increase in some morphological abnormality in endometriosis oocytes, mainly by a presence of endometrioma, the oocyte quality scores Average Oocyte Quality Index (AOQI) and metaphase II oocyte morphological scoring system (MOMS) did not differ between endometriosis and control patients (Robin *et al.*, 2021).

However, the critical question remains whether these alterations in oocyte quality translate into impaired developmental competence. A recent retrospective study including 248 patients with endometriosis, matched by age, stimulation protocol, treatment date, and anti-Müllerian hormone (AMH) levels to 248 controls, found no significant differences in the number of oocytes retrieved, 2PN embryos, blastocysts, or high-quality blastocysts (Invernici *et al.*, 2022). Furthermore, the euploidy rate appears unaffected by endometriosis, as demonstrated by an analysis of 1,873 biopsied embryos from 261 patients, which showed no differences between endometriosis and male factor infertility groups. Clinical pregnancy rates following euploid embryo transfers were also comparable (Bishop *et al.*, 2021). Conversely, a larger retrospective matched cohort study encompassing 3,071 IVF cycles reported that endometriosis negatively influences pregnancy outcomes, being associated with lower cumulative birth rates and diminished oocyte and embryo quantity and quality. This study additionally revealed increased macrophage infiltration and an imbalance between proand anti-inflammatory cytokines in follicular fluid from patients with endometriosis, indicating an altered follicular microenvironment as a potential mechanism (Zhou *et al.*, 2022). Nevertheless, the meta-analyses available to date have not identified significant differences in total or good quality blastocyst numbers, nor in clinical pregnancy or live birth rates between women with and without endometriosis or endometrioma undergoing ART, suggesting no adverse impact on oocyte competence (Qu *et al.*, 2022; Gayete-Lafuente *et al.*, 2024).

Although there is evidence supporting the impact of endometriosis on oocyte quality, its effect on oocyte competence remains less well defined, with most available data suggesting no significant impairment. It is also important to acknowledge that the lack of consistent evidence for reduced oocyte competence in cohort studies and meta-analyses may partly reflect the heterogeneity of the study populations, including differences in disease phenotype, stage, prior surgical history, and clinical management. Nevertheless, it cannot be excluded that oocyte competence may indeed be affected in specific phenotypes or in advanced stages of the disease, such as stage III and IV endometriosis.

## BESIDES OOCYTE QUALITY AND COMPETENCE: THE IMPACT OF ENDOMETRIOSIS ON OVARIAN RESERVE

In addition to oocyte quality and competence, endometriosis may also influence the number of oocytes retrieved. In theory, endometriosis may impair ovarian reserve through chronic systemic inflammation and direct disruption of ovarian function, contributing to reduced follicular recruitment and ovulatory alterations (Cobo *et al.*, 2021).

A meta-analysis, including 26 studies, confirmed that women with endometriosis produce fewer oocytes; however, the analysis did not control for the bias of prior surgical intervention, which limits the causal interpretation of the isolated role of endometriosis (Qu *et al.*, 2022). On the other hand, a recent meta-analysis demonstrates that ovarian endometriosis, endometriomas, reduce both the total oocyte and the number of MII, showing no significant differences in fertilization or blastulation rates (Gayete-Lafuente *et al*., 2024).

As reviewed by La Marca *et al*. (2025), the reduction in ovarian reserve observed in women with endometriomas may result from elevated concentrations of proinflammatory cytokines, proteolytic enzymes, and oxidative stress agents within the endometrioma fluid. These harmful substances can infiltrate the adjacent healthy ovarian cortex, causing damage or loss of primordial follicles and leading to a subsequent decline in AMH levels. Some authors have proposed that, in addition to inflammation and oxidative stress, an increased activation of the PI3K-AKT pathway associated with endometriosis may lead to the premature activation of dormant primordial follicles, thereby accelerating follicular depletion and contributing to a reduced ovarian reserve. According to this hypothesis, the local inflammatory response and subsequent fibrosis at endometriotic sites, alongside Wnt signaling dysregulation, could impair ovarian vascularization, nutrient supply to developing follicles and promote cell death and follicular atresia (Demirel *et al.*, 2001; Takeuchi *et al.*, 2019; Fan *et al.*, 2023).

Furthermore, the presence of large endometriomas may impose mechanical stress on ovarian tissue, further compromising the ovarian reserve. Moreover, a pragmatic study by Cobo et al. (2021) showed that the most direct and measurable detrimental impact of endometriomas on ovarian reserve appears to be associated with cystectomy, particularly when performed in young women, showing lower cumulative live birth rates than nonoperated women in age-matched groups.

To date, available evidence suggests that the impact of endometriosis lies more in the quantity of oocytes retrieved rather than in their quality or competence. As a consequence of a reduced number of oocytes, fewer blastocysts become available for transfer, ultimately leading to a lower cumulative live birth rate in patients with endometriosis as shown in Zhou *et al*. (2022).

Given this quantitative decline, fertility preservation has been increasingly discussed as a potential strategy in the management of endometriosis. The ESHRE guideline (2022) recommends that, in cases of extensive ovarian endometriosis, clinicians should discuss fertility preservation with affected women, as its actual benefit in this population remains uncertain (Becker *et al.*, 2022). In contrast, the most recent ASRM Committee Opinion on endometriosis and infertility does not specifically address fertility preservation, as its focus is mainly on the management of existing infertility rather than on preventive strategies (Practice Committee of the American Society for Reproductive Medicine, 2012). Despite the absence of formal recommendations from reproductive societies or clinical guidelines, fertility preservation has been proposed for patients over 35 years, those with bilateral or unilateral endometriomas (prior to surgery), diminished ovarian reserve, a history or risk of multiple surgeries, large endometriomas (>4 cm), severe endometriosis (stages III and IV), low endometriosis fertility index (EFI) scores, and a low probability of natural conception following surgery (La Marca *et al.*, 2025).

## ENDOMETRIAL ALTERATIONS IN ENDOMETRIOSIS: THEIR CLINICAL RELEVANCE

One of the major challenges in reproductive physiology, and consequently in IVF, is to understand the key factors that determine successful embryo implantation and to elucidate the complex cross-talk between the embryo and the endometrium, often referred to as the ‘black box’ of implantation. Implantation failure, particularly involving euploid embryos, remains one of the most enigmatic and complex issues in assisted reproductive technologies (Cimadomo *et al.*, 2023). Therefore, any condition that might impair endometrial receptivity is a consistent focus of investigation, as is the case with endometriosis.

In endometriosis, alterations in molecular pathways, such as those related to epigenetic regulation, immune and inflammatory responses, hormonal signaling, and epithelial-mesenchymal transition, have been suggested to impair embryo endometrium cross-talk, potentially contributing to implantation failure (Guo *et al.*, 2023). Moreover, endometriosis is commonly associated with an increased inflammation-related in-situ production of estradiol and progesterone resistance (de Ziegler *et al.*, 2010).

Single-cell transcriptomic analyses have revealed that even the eutopic endometrium of women with endometriosis displays distinct cellular and molecular alterations compared to healthy controls. These changes involve transcriptional reprogramming and structural remodeling of epithelial and stromal cells, as well as dysregulation of immune related pathways (Fonseca *et al.*, 2023). Likewise, a proinflammatory immune profile in the eutopic endometrium of women with endometriosis is affected, with increased CD8+ T cells linked to a higher risk of infertility (Wu *et al.*, 2021). Several studies have reported alterations alterations in the endometrium, ranging from dysregulation of immune pathways to epigenetic factors (Hromadnikova *et al.*, 2022; Shi *et al.*, 2025). Nonetheless, while several studies have reported changes in the endometrial quality, it remains to be clarified whether such alterations compromise endometrial competence, the ability to support implantation and achieve a live birth.

One of the most widely discussed studies on this topic is the meta-analysis published in JAMA Network Open by Paffoni *et al*. (2024) which evaluated live birth rates in women with endometriosis who received donor oocytes, as it provides robust data on endometrial receptivity by excluding the potential confounding factor of impaired embryo quality in these patients. This systematic review and meta-analysis included data from four individual studies (two prospective and two retrospective) as well as from two major IVF registries: the Human Fertilization and Embryology Authority (HFEA, UK) and the Society for Assisted Reproductive Technology (SART, USA). In total, 7,212 donor oocyte cycles derived from the studies were examined, in addition to 162,082 cycles reported by the registries (137,182 from SART and 24,900 from HFEA). The adjusted live birth outcome showed a small, but statistically significant, difference in patients with endometriosis (OR, 0.89; 95% CI, 0.81-0.97), observed exclusively in the registry data, not in the meta-analysis of prospective or retrospective studies. The authors conclude that there appears to be a minimal reduction in live birth rates among recipients with a history of endometriosis undergoing ART cycles with donor oocytes. They also suggest that a marginal impairment of uterine receptivity may be present in women affected by endometriosis. However, whether this impairment is related to comorbidities or confounding factors remains unclear and should be further investigated in future studies.

These findings are consistent with those reported reported by Bishop et al. (2021), which showed no statistically significant differences in clinical outcomes following single euploid embryo transfer among patients with endometriosis, male factor infertility, and PGT-M cycles, as well as with a recent meta-analysis on clinical outcomes in endometriosis patients undergoing IVF (Qu *et al.*, 2022). On the other hand, an interesting meta-analysis identified an approximately 12% reduction in live birth rates following ART specifically among patients with stage III-IV endometriosis, highlighting that the phenotypic expression and clinical impact of the disease may vary according to its stage and subtype (Horton *et al.*, 2019).

Regarding cumulative live birth rates, when analyses control for confounding factors such as the number of oocytes retrieved and the number and quality of embryos obtained, it becomes more appropriate to assess the potential impact of endometriosis on endometrial receptivity. A recent matched cohort study, which included 100 women with endometriosis and 100 controls matched for age, as well as the number and quality of blastocysts, reported comparable cumulative live birth rates between the groups (Casalechi *et al.*, 2023). These findings support the hypothesis that endometriosis does not significantly compromise endometrial receptivity in affected individuals.

This apparent discrepancy between molecular alterations and preserved clinical outcomes may reflect a certain degree of endometrial resilience, in which compensatory mechanisms maintain implantation capacity despite local disturbances. Likewise, it is plausible that compensatory molecular pathways or redundant mechanisms involved in implantation and early placentation mitigate the functional impact of the transcriptomic and proteomic disturbances observed in endometriosis.

Despite the lack of robust evidence supporting a negative impact of endometriosis on clinical pregnancy or live birth rates, a recent meta-analysis highlights that severe forms of the disease (stage III-IV and deep endometriosis) represent significant risk factors for placenta previa and preterm birth (Busnelli *et al*., 2024), suggesting that the impact of endometriosis may manifest later in pregnancy.

## LESSONS FROM THERAPEUTIC INTERVENTIONS DURING ART

The appropriate timing, indication, and type of treatment for endometriosis in infertile patients remain topics of ongoing debate. Some authors have proposed structured management approaches to guide clinical decision-making in this population (Di Spiezio Sardo *et al.*, 2025). In summary, for women under 35 years of age experiencing pelvic pain, conservative surgical treatment may be considered, and if spontaneous pregnancy does not occur within one year postoperatively, IVF (with or without prior medical therapy) may be indicated. For women over 35 years of age with pain, or for asymptomatic women, proceeding directly to IVF with possible pre-treatment using medical therapy is generally recommended. Furthermore, in cases of repeated IVF failure, surgical intervention may be considered (Di Spiezio Sardo *et al.*, 2025).

For selected patients, hormonal or surgical treatment may precede IVF or embryo transfer. GnRH agonists have demonstrated anti-inflammatory, anti-angiogenic, and pro-apoptotic effects beyond their hypoestrogenic mechanism (Khan *et al.*, 2010), while progestins may improve the endometrial environment by reducing inflammatory cytokines and oxidative stress (Tamura *et al.*, 2019). However, evidence remains inconsistent. The most recent Cochrane meta-analysis, including eight RCTs, found low-certainty evidence and concluded that prolonged GnRH agonist pretreatment does not clearly improve pregnancy or live birth rates (Georgiou *et al.*, 2019). Similarly, a network meta-analysis of nine RCTs found no benefit of hormonal pretreatment before ovarian stimulation, with higher live birth rates observed in women who did not receive pretreatment (Riemma *et al.*, 2025).

Consistently, the ESHRE guideline states that neither hormonal nor surgical interventions should be routinely used before IVF to improve live birth rates (Becker *et al.*, 2022). Surgery may be considered only to alleviate pain or improve follicle accessibility in specific cases, such as endometrioma or deep lesions. Overall, the absence of consistent benefit from pre-IVF interventions reinforces the notion that endometriosis exerts a limited direct impact on ART outcomes.

## CONCLUSION: WEIGHING THE EVIDENCE: VERDICT YET TO BE REACHED

Most of the current evidence linking endometriosis and (in)fertility originates from ART cycles, which may not fully represent physiological reproductive conditions. Furthermore, many studies reporting clinical pregnancy rates aggregate data from women attempting natural conception and those undergoing ART, across varying stages of endometriosis, with or without comorbidities such as adenomyosis, and under highly heterogeneous treatment protocols, all factors that complicate the interpretation of definitive conclusions.

**Figure 2:**
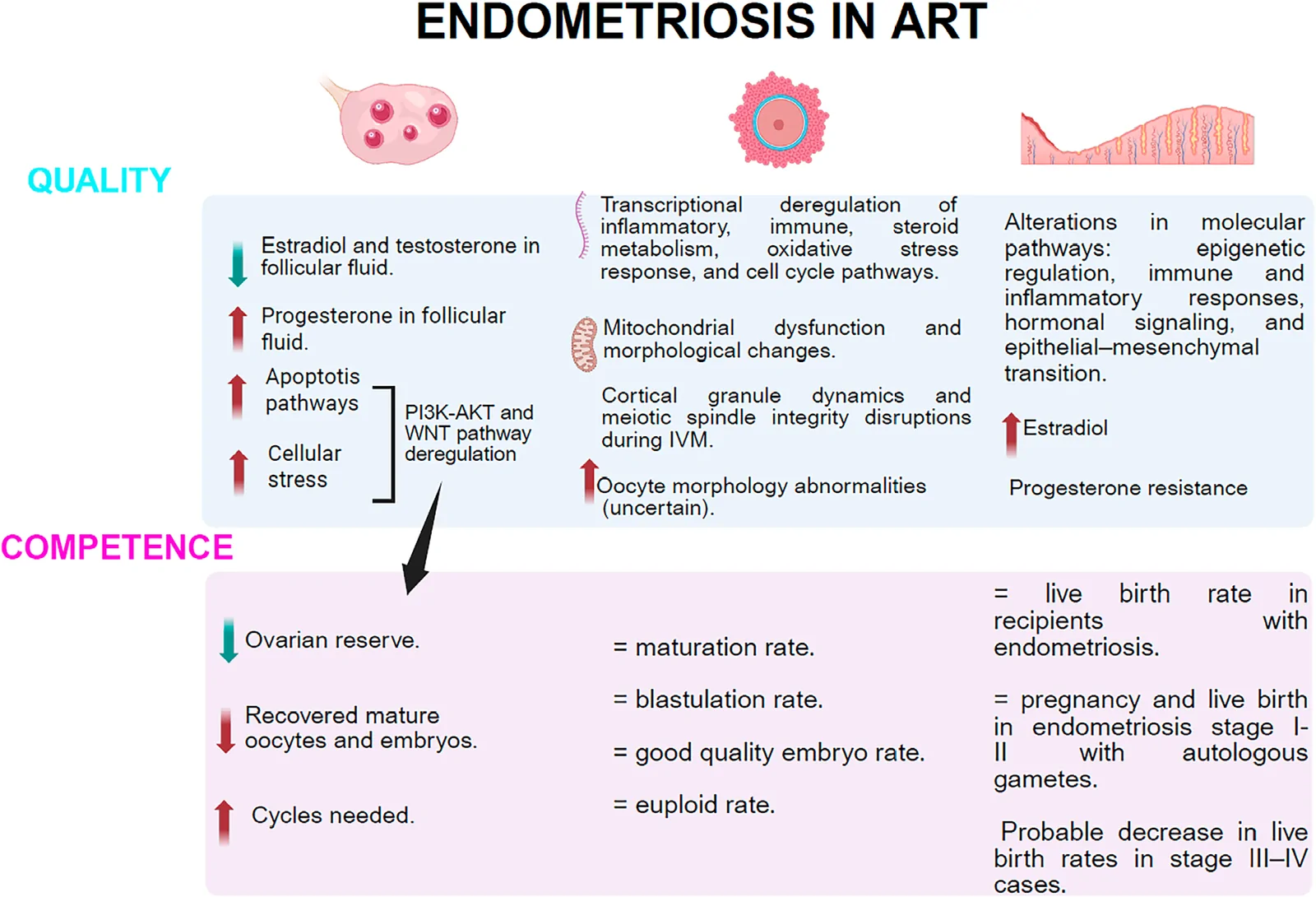
Schematic representation of the possible mechanisms by which endometriosis affects reproductive outcomes in assisted reproductive technology (ART) at multiple levels. The upper panel depicts alterations in ovarian, oocyte, and endometrial quality, including hormonal imbalances, oxidative stress, apoptosis, mitochondrial and spindle defects, and transcriptional dysregulation. The lower panel illustrates the downstream consequences on ovarian reserve, embryo development, and clinical outcomes, such as reduced numbers of mature oocytes and embryos and compromised embryo developmental and implantation rates.

Although it is well established that endometriosis alters the reproductive tract milieu and, consequently, the local microenvironment, the precise repercussions of these alterations on gamete and endometrial function remain incompletely understood (Figure 2).

Undeniably, endometriosis can lead to infertility when pelvic adhesions cause tubal obstruction or when severe pain interferes with sexual intercourse. In the context of ART. However, large-scale studies have not consistently demonstrated a detrimental effect of endometriosis on reproductive competence, although more severe phenotypes (stages III-IV) may be associated with greater adverse outcomes.

In summary, until new and robust evidence emerges to challenge current assumptions, reasonable doubt persists regarding the true extent to which endometriosis influences fertility outcomes in IVF cycles. It may now be time to broaden the investigative lens from a micro-level focus on cellular quality to a more integrated perspective that also encompasses cellular competence. Future research should employ matched-cohort designs that control for the type and stage of endometriosis, as well as for oocyte yield, to better isolate disease-specific effects. Ideally, these studies should integrate molecular and clinical analyses, linking oocyte quality assessments with measures of developmental and clinical competence, such as live birth and cumulative live birth rates. Moreover, studies using donor oocytes may help clarify whether endometrial receptivity is genuinely impaired in women with endometriosis. Collectively, such approaches may ultimately determine whether endometriosis compromises oocyte or embryonic competence and/or endometrial receptivity, thereby providing a more comprehensive understanding of its true impact on reproductive outcomes.
